# Metabolic Syndrome and Skin Diseases

**DOI:** 10.3389/fendo.2019.00788

**Published:** 2019-11-20

**Authors:** Yu Hu, Yun Zhu, Ni Lian, Min Chen, Andrzej Bartke, Rong Yuan

**Affiliations:** ^1^Jiangsu Key Laboratory of Molecular Biology for Skin Diseases and Sexually Transmitted Infections, Institute of Dermatology, Chinese Academy of Medical Sciences and Peking Union Medical College, Nanjing, China; ^2^Department of Internal Medicine, Southern Illinois University School of Medicine, Springfield, IL, United States

**Keywords:** metabolic syndrome, skin diseases, insulin resistance, adipokine, proinflammatory cytokine

## Abstract

The increasing prevalence of Metabolic syndrome (MetS) is a worldwide health problem, and the association between MetS and skin diseases has recently attracted growing attention. In this review, we summarize the associations between MetS and skin diseases, such as psoriasis, acne vulgaris, hidradenitis suppurativa, androgenetic alopecia, acanthosis nigricans, and atopic dermatitis. To discuss the potential common mechanisms underlying MetS and skin diseases, we focus on insulin signaling and insulin resistance, as well as chronic inflammation including adipokines and proinflammatory cytokines related to molecular mechanisms. A better understanding of the relationship between MetS and skin diseases contributes to early diagnosis and prevention, as well as providing clues for developing novel therapeutic strategies.

## Introduction

Metabolic syndrome (MetS), first described in 1988 by GM Reaven as “syndrome X,” is a group of abnormalities which includes abdominal obesity, hypertension, insulin resistance, and dyslipidemia (1, 2). It is estimated that about one-quarter to one-third of the world's population is affected by MetS (3). Growing evidence from clinical studies suggests that there exists a strong connection between MetS and skin diseases (4, 5). However, mechanistic insights regarding the relationship of metabolic syndrome and skin diseases are notably lacking. In this review, we will summarize the epidemiological evidence for the association between MetS and skin diseases, as well as discuss the potential common mechanisms underlying MetS and skin diseases.

## Epidemiological Evidence of the Connection Between MetS and Skin Diseases

### Psoriasis

Psoriasis is one of the most common chronic inflammatory skin diseases, with the prevalence ranging from 0.51 to 11.43% in adults and from 0 to 1.37% in children (6). The association between psoriasis and MetS has been investigated in numerous studies. Two recent systematic reviews demonstrated that psoriasis patients have an increased risk for MetS (7, 8). Singh and his colleagues (7) reviewed 17 articles involving 3,791 psoriasis patients in a group of 28,939 participants from January 1946 to June 2016, and reported a higher prevalence of both MetS and the individual components of MetS including abdominal obesity, elevated fasting plasma glucose, and high blood pressure in the psoriasis patients. The same group then updated the data from June 2016 to January 2017 and conducted a meta-analysis. The meta-analysis included 35 studies with 1,450,188 participants, of whom 46,714 were psoriasis patients. They found that, compared to the general population, psoriasis patients had a higher prevalence of MetS with the pooled odds ratio (OR) of 2.14 (95% CI 1.84–2.48) (9). Another systematic review and meta-analysis investigated 14 papers including 25,042 psoriasis patients and also found that 31.4% psoriasis patients had MetS with the pooled OR of 1.42 (95% CI, 1.28–1.55) (8). When examining the association between disease severity and MetS, a dose-dependent relationship between Psoriasis Area and Severity Index (PASI) and the prevalence of MetS was observed (9). The adjusted ORs for MetS in severe, moderate, and mild psoriasis were 1.98, 1.56, and 1.22, respectively. Additionally, a recent population-based study in United Kingdom also supported a positive correlation between PASI and the prevalence of MetS, although it was not significant (OR = 1.18, 95% CI, 0.97–1.44, *p* = 0.099) (10).

#### Acne Vulgaris

Acne vulgaris is an inflammatory disorder of the pilosebaceous unit which affects many adolescents and young adults. A cross-sectional study was performed by comparing the metabolic conditions in 100 male acne patients and 100 male controls. It showed that the prevalence of metabolic syndrome tends to be higher in acne patients (17%) compared with controls (9%) (*p* = 0.09), and also that prevalence of insulin resistance is significantly higher in acne patients (22%) than controls (11%) (*p* = 0.03) (11). Another study of 243 acne patients and 156 controls also found that fasting insulin levels were significantly higher in acne patients (12). However, a recent nationwide study in Israel showed that acne and metabolism have a complicated relationship. In this study, including 600,404 adolescents, the researchers found that overweight or obesity is inversely associated with acne in a dose-dependent manner, suggesting that excessive body mass index (BMI) has a protective effect against acne (13). The possible mechanism of the protective effect is increased aromatase activity, which leads to a greater conversion of testosterone to estradiol (14). Estrogen is known to act by anti-androgenic effect, inhibiting sebum secretion and attenuating proinflammatory cytokine activity (15). Moreover, obesity is reported to reduce the conversion of testosterone to the more physiologically active dihydrotestosterone by suppressing the activity of 5-α reductase-II (16).

#### Hidradenitis Suppurativa

Hidradenitis suppurativa (HS) is a chronic inflammatory skin disease involving the follicular portion of folliculopilosebaceous units with an estimated prevalence from 0.05 to 4.10% (17). The accumulating data have shown a positive association between HS and MetS (18–20). A cross-sectional study in Israel, which included 3,207 HS patients and 6,412 controls, revealed that HS was significantly associated with MetS (OR 1.61, 95% CI 1.36–1.89) (18). When comparing the prevalence of MetS in both hospital HS and population HS groups vs. non-HS groups, the ORs were 3.89 (95%CI, 1.90–7.98) for hospital HS and 2.08 (95%CI, 1.61–2.69) for population HS (19). Another retrospective review, which enrolled 366 HS patients and 366 controls of all races, also indicated that patients with HS might have a greater risk for MetS. They found the prevalence of MetS in HS patients (50.6%) was significantly higher than controls (30.2%, *p* = 0.001) (20).

### Androgenetic Alopecia

Androgenetic alopecia (AGA) is a common type of progressive non-scarring hair loss in both men and women. The incidence of AGA varies by race and age. Around 30% of Caucasian men will have AGA at the age of 30, 50% at the age of 50, and 80% at the age of 70. The prevalence of AGA in Caucasian women is about 19%. Asians are less affected by AGA than both male and female Caucasians (21). Numerous studies have reported a strong association between AGA and MetS (22–26), although one case-control study (27) in Turkey of 74 male AGA patients and 42 controls, found that there was no difference in the rate of MetS. However, another case-control study by Dharam and his colleagues found a higher prevalence of MetS in AGA patients (53%) than controls (17%) (*P* < 0.001) (26). Similar findings were reported in a hospital-based cross-sectional study in India, in which 19 of 85 (22.4%) AGA patients were affected with MetS compared with 8 of 85 (9.4%) controls (*p* = 0.021) (22). However, when analyzing the relationship between each component of MetS and AGA, different results were reported. A survey conducted in Taiwan noted that high-density lipoprotein cholesterol (HDL-C) (OR 2.36, 95% CI 1.41–3.95; *P* = 0.001) was the most significant factor associated with AGA (25). Another case-control study reported that waist circumference (>102 cm) was the most significant risk factor for AGA patients to develop MetS with the value of 1.25 (95% CI = 1.10–1.42, *P* < 0.001) (28). Similarly, the research in female AGA patients revealed waist circumference (OR 5.6, 95% CI 2.2 −13.9, *P* = 0.0002) and hypertension (OR 3.5, 95% CI 1.3–8.9, *P* = 0.008) were the most important factors for AGA (29).

### Acanthosis Nigricans

Acanthosis nigricans (AN) is a common skin disorder characterized by hyperpigmented, velvety patches and plaques involving the intertriginous areas. The prevalence of AN varies among different ages and ethnicities, reaching 25% in the general population and even over 60% in overweight and obese children (30). There is solid evidence for the existence of a link between AN and MetS (31–33). For overweight and obese women, the incidence of MetS is significantly higher in AN patients (60%) than controls (37.6%) (*p* = 0.0001)(31). A case-control study, which included 100 children who were overweight or obese, showed that 73% of them had a diagnosis of MetS with a strong association to AN (OR 1,872 [95% CI: 112.9–31,028]) (32). Another cross-sectional study also found that AH in overweight and obese children might be a clinical indicator of MetS, with the elevation of body fat, blood pressure, insulin, and homeostasis model assessment index (33). Furthermore, a 2-year multicenter case-control study including 123 patients at the age of 38.83 ± 8.62 years in India, found that facial AN was strongly related to impaired glucose tolerance, increased waist–hip ratio (WHR), and increased BMI. The authors suggest that the facial AN might be a potential clinical marker of MetS (34).

### Atopic Dermatitis

Atopic dermatitis (AD) is a chronic pruritic inflammatory dermatosis which is commonly associated with other atopic disorders including food allergies, asthma, hay fever, and allergic rhinitis. The worldwide prevalence of AD is approximately 15–20% in children and 1–3% in adults (35). The association between AD and MetS has not been fully clarified. A cross-sectional study in 5,007 Korean adults reported that MetS (OR 2.92, 95% CI 1.49–5.73), central obesity (OR 1.73, 95% CI 1.09–2.75), and hypertriglyceridemia (OR 2.20, 95% CI 1.19–4.05) correlated positively with AD in women (36). However, unlike the psoriasis cohort, the prevalence ratios (PR) for MetS-associated components including hypertension (PR 0.83, 95% CI 0.81–0.85), hyperlipidemia (PR 0.94, 95% CI 0.91–0.95), and diabetes (PR 0.82, 95% CI 0.80–0.86) showed no difference in AD patients compared to non-AD patients (37). Moreover, a recent systematic review, which included 14 studies evaluating the relationship between AD and MetS, reported that the association between AD and MetS was not causal. The associations between hypertension, hyperglycemia, cholesterol levels, and AD all remain unclear. According to the systematic review, central obesity is the only component that correlates positively with AD (38). This phenomenon was also reported in a case-control study and a systematic review. These two studies both reported that obesity is associated with increased prevalence and severity of AD (39, 40).

## Mechanisms of MetS and Skin Diseases

Although the exact mechanisms of the association of MetS and skin diseases remain vague, it is of great importance to identify the potential common pathways behind MetS and associated skin diseases in order to develop novel therapeutic strategies. Of all the potential pathological mechanisms of MetS, emerging evidence suggests that impaired insulin signaling and increased insulin resistance, as well as elevated status of chronic inflammation, are major risk factors that may induce skin diseases (41–43).

### Insulin Signaling and Insulin Resistance

**Figure 1:**
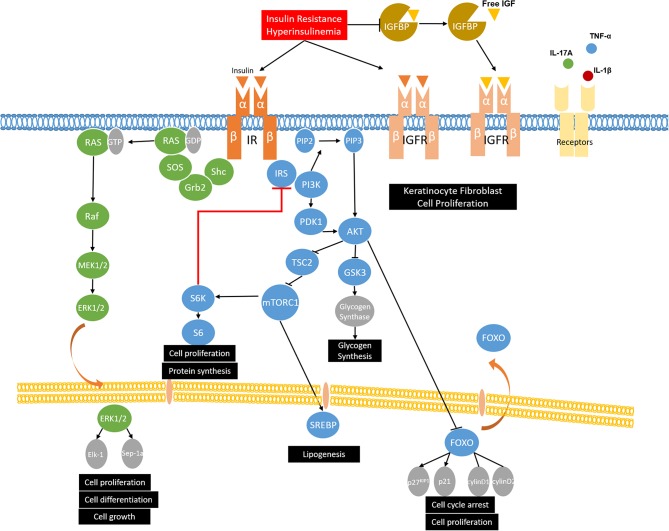
The role of insulin signaling and insulin resistance in MetS and skin diseases. Insulin binds to insulin receptor (IR) and phosphorylates insulin receptor substrates (IRS-1, IRS-2) and Shc, which activates the two main insulin signaling pathways: the phosphatidylinositol-3-kinase (PI3K)/Akt pathway and the mitogen-activated protein kinases (MAPK)/Ras pathway. Under insulin resistance and compensatory hyperinsulinemia condition, insulin not only binds to IR, but also binds to IGF-receptors and stimulates the proliferation of keratinocytes and fibroblasts. Moreover, insulin resistance and compensatory hyperinsulinemia could decrease the expression of insulin-like growth factor binding proteins (IGFBPs), thus increasing biological active IGF-1 and resulting in the development of hyperkeratosis and papillomatosis, which are demonstrated as the possible pathogenesis of acanthosis nigricans. As for acne vulgaris and hidradenitis suppurativa, western diet and puberty increase PI3K/Akt signaling and activate mTORC1. mTOR causes the serine/threonine phosphorylation of IRS by activating S6K and reduces its ability to be phosphorylated on tyrosine residues, which results in invalid insulin signaling and insulin resistance. mTORC1 also promotes lipid synthesis via activating the transcription factor sterol regulatory element binding protein 1 (SREBP-1) and inducing the expression of acetyl CoA carboxylase (ACC), which is the rate-limiting enzyme of fatty acid synthesis. In psoriasis, overactivation of PI3K and Akt phosphorylates FOXO and leads to its nuclear export, thus promoting cell proliferation by suppressing its function of activating cell cycle inhibitors (p27KIP1 and p21) and repressing cell cycle activators (cyclin D1/D2), which contributes to proliferation of keratinocytes. Moreover, in psoriatic condition, growth factors and relevant cytokines (IL-17A, TNF-α, and IL-1β) in psoriasis activate mTOR and then promote keratinocyte hyperproliferation and inhibiting differentiation. In the MAPK/Ras pathway, ERK1/2 could activate upstream MEK, reduce Akt phosphorylation, and contribute to insulin resistance. Furthermore, p-ERK1/2 have been identified to be increased in psoriatic skin, which results in the abnormal and proliferation and differentiation of keratinocytes.

Insulin, secreted by the beta cells of the pancreas, is involved in glucose homeostasis, lipid metabolism, and anabolic processes by activating the insulin signaling pathways. Insulin initiates its function by binding to insulin receptor (IR). After stimulation, IR is activated via autophosphorylation and leads to the phosphorylation and activation of the downstream substrates, such as insulin receptor substrates (IRS-1, IRS-2) and Shc. Then the phosphorylated tyrosine residues of IRS and Shc can activate the two main insulin signaling pathways: the phosphatidylinositol-3-kinase (PI3K)/Akt pathway and the mitogen-activated protein kinases (MAPK)/Ras pathway (44). In the PI3K/Akt pathway, the phosphorylated tyrosine residues of IRS activate PI3K and phosphorylates phosphatidylinositol (4, 5)-bisphosphate (PIP2), leading to the formation of phosphatidylinositol 3–5 three phosphoric acid (PIP3). Then, PIP3 facilitates the phosphorylation of Akt by 3-Phosphoinositide-dependent protein kinase-1 (PDK1). Once activated, Akt phosphorylates tuberous sclerosis complex 2 (TSC2) and suppresses its inhibitory effect on mechanistic target of rapamycin complex 1 (mTORC1), thus upregulating the activity of mTORC1, which regulates protein synthesis and cell growth (45). The activated Akt may also phosphorylate the downstream target FOXO1, leading to nuclear export and inhibiting FOXO1 transcriptional activity (46). FOXO1 is involved in adipogenesis and gluconeogenesis by controlling the expression of lipogenic and gluconeogenic genes, as well as cell proliferation and apoptosis (47, 48). The other branch of the insulin signaling pathway is the MAPK/Ras pathway, which is mediated by the growth factor binding protein 2 (Grb2). Active Ras then interacts with, and leads to, the activation of downstream Raf and MAP kinases ERK1/ERK2. After activation, ERK1/2 regulates gene expression, cell proliferation, differentiation, and cell growth by translocating to the nucleus and catalyzing the phosphorylation of transcription factors, such as Elk-1 and Sep-1a (Figure 1) (49, 50).

Insulin resistance is defined as the reduced responsiveness of target tissues to normal insulin levels and is widely accepted as the primary mechanism of the pathophysiology of MetS. Though many cell types express insulin receptors, liver, skeletal muscle, white adipose tissue, and brain are the main tissues responsible for glucose homeostasis (51). In insulin resistance, the cells are resistant to insulin and fail to maintain the blood glucose level. To achieve a normal blood glucose level, pancreatic β-cells secret excessive insulin, which leads to hyperinsulinemia (52). With further resistance to insulin, the function of β-cells is impaired and becomes insufficient to maintain the normal blood glucose range, resulting in hyperglycemia. A high plasma glucose level also results from an impaired inhibitory action of insulin on hepatic glucose production, a reduction of glycogen synthesis in hepatocytes and the inability of skeletal muscle and adipocytes to take up glucose (53). The possible pathophysiologic mechanisms that provoke insulin resistance in MetS include defective insulin signaling, impaired glucose disposal, lipotoxicity, and inflammatory cytokines.

The two main target cell types in skin diseases, keratinocytes and fibroblasts, both have insulin receptors and IGF receptors. Insulin has been reported to cross the dermal-epidermal junction to affect keratinocytes (54). The growth-promoting effects of insulin in keratinocytes and fibroblasts rely on the following mechanisms. First, insulin resistance and compensatory hyperinsulinemia decrease the expression of insulin-like growth factor binding proteins (IGFBPs) (55). IGFBPs bind to IGFs and extend their half-life, thereby controlling the transport of IGFs to target tissues and regulating the level of circulation IGFs (56). The high levels of free IGF-1 lead to the proliferation and differentiation of fibroblasts and keratinocytes (57). IGFBPs also exert antiproliferative effects by blocking the interaction of IGF and IGF-receptors because the affinity of IGFs for IGFBPs is similar to that of the IGFs and IGF-receptors (58). Second, under low insulin concentrations, insulin is more likely to bind to its classic receptor and then regulate glucose metabolism. Under insulin resistance and compensatory hyperinsulinemia condition, insulin might have a potential to bind to IGF-receptors and stimulate the proliferation of keratinocytes and fibroblasts (59). However, it is reported that the affinity of insulin binding to IGF-receptors is approximately 2000-fold lower than binding to IR (60). Thus, we propose that the compensatory hyperinsulinemia exceeds the binding capacity of IR, allowing the insulin to bind to IGF-receptors. These two mechanisms have been proposed as the possible pathogenesis of acanthosis nigricans. In brief, hyperinsulinemia in acanthosis nigricans patients not only can increase the binding of insulin and IGF-receptors, but also can reduce IGFBPs, thus increasing biologically active IGF-1, and resulting in the development of hyperkeratosis and papillomatosis (54, 59).

As for acne vulgaris and hidradenitis suppurativa, mechanistic target of rapamycin complex 1 (mTORC1) is the key element that associates with insulin signaling and insulin resistance (61–64). Western diet and puberty, which are the two main risk factors of acne vulgaris, increase insulin/IGF-1 signaling and activate Akt. Active AKT stimulates mTORC1 activity by inhibiting TSC2. Ribosomal S6 kinase 1(S6K1), which is a downstream target of mTORC1, contributes to the serine/threonine phosphorylation of IRS, thus inducing insulin resistance by antagonizing tyrosine phosphorylation (65). mTORC1 also promotes lipid synthesis via activating the transcription factor sterol regulatory element binding protein 1 (SREBP-1) and inducing the expression of acetyl CoA carboxylase (ACC), which is the rate-limiting enzyme of fatty acid synthesis (66, 67). In acne vulgaris, overexpression of free fatty acids such as sapienic acid, oleic acid, and palmitic acid promotes propionibacterium acnes biofilm formation, stimulates T helper cell 17 (Th17)-driven inflammation, and disturbs follicular keratinization and barrier function (68). The role of mTORC1 in hidradenitis suppurativa is related to its function in cell proliferation, especially in sebaceous glands and keratinocytes. Taken together, Western diet and puberty, in the conditions of acne and hidradenitis suppurativa, increase insulin signaling and activate mTORC1. Increased mTORC1 activity then contributes to insulin resistance, which means acne vulgaris and hidradenitis suppurativa are not only skin diseases but also are more like systemic diseases.

A recent study has reported that the PI3K/Akt pathway is overexpressed in peripheral blood mononuclear cells (PBMCs) and keratinocytes of psoriasis patients (69, 70). Compared with normal skin and uninvolved psoriatic skin, the psoriatic skin has a repressed expression of FOXO1 (71). As described above, Akt phosphorylates FOXO and leads to its nuclear export, thus promoting cell proliferation by suppressing its function of activating cell cycle inhibitors (p27KIP1 and p21) and repressing cell cycle activators (cyclin D1/D2) (72). Thereby, overactivation of PI3K and Akt and the downregulation of FOXO contribute to proliferation of keratinocytes, which might be the potential role of PI3K/Akt signaling in psoriasis. Another downstream molecule of PI3K/Akt pathway, mTOR and its phosphorylation, have also been reported to be hyperactivated in lesional psoriatic skin. Buerger and his colleagues found that mTOR is activated throughout the whole epidermis of psoriatic skin, particularly in the basal layer, and the downstream molecules S6K-1 and ribosomal protein S6 are only activated in suprabasal layers in lesional skin (73). Their group also proposed a model where mTORC1 functions as a central switch between keratinocyte proliferation and differentiation (74). In the basal layer of healthy skin, the mTORC1 signaling pathway controls proliferation and blocks differentiation. When keratinocytes leave the basal layer, the mTORC1 pathway is switched off and this leads to the differentiation. Nevertheless, in the psoriatic condition, the mTORC1 signaling pathway is permanently switched on and results in epidermal hyperplasia by promoting hyperproliferation and inhibiting differentiation of keratinocytes (74, 75). Recent studies also revealed that inflammatory cytokines are associated with the activation of the mTORC1 signaling cascade. Patel and his colleagues found that tumor necrosis factor α (TNF-α) can activate mTOR and then stimulate IL-6, CXCL8, and VEGF secretion from both HaCat cells and primary human keratinocytes (76). Another study reported IL-17A-mediated inflammatory stimulation in keratinocytes activates PI3K/AKT/mTOR signaling and inhibits autophagy (70). Consistent with the previous studies, the Buerger laboratory found that inflammatory cytokines, such as IL-1β, IL-17A, and TNF-α, promote the mTOR pathway via PI3K signaling and lead to enhanced proliferation in psoriatic keratinocytes (77). Then, growth factors and relevant cytokines (IL-17A, TNF-α, and IL-1β) in psoriasis activate mTOR and promote keratinocyte hyperproliferation and inhibit differentiation, which might be the mechanism underlying the PI3K/Akt pathway and psoriasis (78). Therefore, we hypothesize that mTOR may function as a key target by connecting psoriasis and MetS through insulin resistance. mTOR causes the serine/threonine phosphorylation of IRS by activating S6K, and reduces its ability to be phosphorylated on tyrosine residues, which results in impaired insulin signaling and insulin resistance (52). Moreover, insulin resistance can exacerbate inactivation of the PI3K/AKT signaling pathway, which is characteristic of metabolic syndrome (79, 80).

Although the main function of the MAPK/Ras pathway was thought to be the regulation of mitogenesis, it has been reported that ERK1/2 can activate upstream MEK, reduce hepatic Akt phosphorylation, and contribute to insulin resistance (80). Furthermore, p-ERK1/2 has been found to be increased in psoriatic skin, which results in the abnormal proliferation and differentiation of keratinocytes (81, 82). ERK phosphorylation can also activate Th17 cells and then promote the secretion of inflammatory cytokines TNF-α, IL-6, and IL-17A, which positively correlate with psoriasis. A recent study reported that ERK inhibitor JSI287 alleviates imiquimod-induced psoriasis-like dermatitis by suppressing the ERK/IL-17 signaling pathway (83).

### Chronic Inflammation

**Figure 2:**
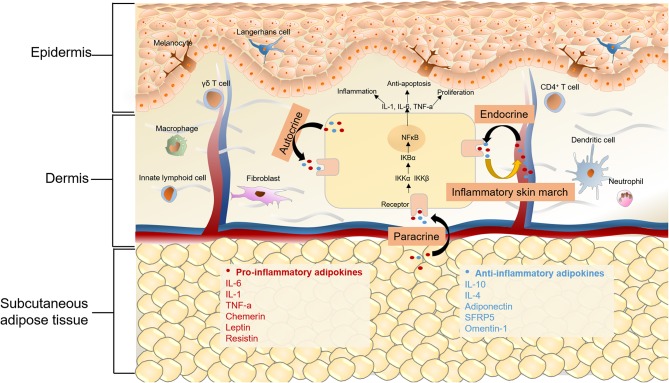
Chronic inflammation in MetS and skin diseases. Adipokines can be classified as pro-inflammatory and anti-inflammatory ones depending on their effects on inflammation. Anti-inflammatory adipokines are represented by adiponectin, secreted frizzled-related protein 5 (SFRP5) and omentin-1, as well as classical cytokines like IL-6, IL-10, IL-4. Chemerin, leptin, resistin and classical cytokines like TNF-α and IL-6 belong to pro-inflammatory adipokines. On one hand, for metabolic syndrome patients, altered adipokines secretion may mediate cutaneous inflammatory response through autocrine, paracrine and endocrine. On the other hand, the “inflammatory skin march” might be another mechanism underlying the connection of MetS and some skin diseases. The cutaneous inflammatory mediators could migrate into the systemic circulation and the release of these proinflammatory cytokines results in the chronic systemic inflammation and MetS. The activation of NFkB signaling pathway is a commom pathway underlying skin diseases and MetS, in the canonical NFkB pathway, the dimer of p65 and p50 is in an inactive state by the inhibition of IκB in cytoplasm. Upon stimulation by various cytokine receptors, IKKs are activated, leading to phosphorylate IκB, which results in their degradation and enables NFκB translocate to the nucleus to induce target gene expression. The activation of NFκB then produces a wide range of chemokines and cytokines, which leads to the formation of a feed forward loop, and establishs a chronic inflammatory environment.

It is evident that MetS is a chronic inflammatory status with increased levels of proinflammatory cytokines, inflammatory biomarkers, and altered adipokines. Liver, intestine, and adipose tissue are the major three sites of the initiators of inflammation in MetS, of which, adipose tissue is vital (84). Because of nutritional excess in MetS, adipose tissue mass expands and adipocytes become hypertrophic, which can lead to decreased insulin sensitivity, hypoxia, altered autophagy, and apoptosis of adipocytes (85). The dysfunction of adipose tissue and impaired condition of adipocytes can also lead to overproduction of proinflammatory cytokines, such as interleukin 6 (IL-6), TNF-α, and C-reactive protein (CRP), thus resulting in macrophage infiltration (41, 42). Adipokines, which are mainly secreted by adipocytes, also play a significant role in MetS. In regard to the relationship between adipocytes and skin diseases, previous studies mostly focused on their role in regulating hair regeneration, wound healing, and skin aging (86, 87). Recently, the secretion of cytokines and adipokines by adipocytes has received increased attention, which might provide a novel angle in understanding the pathological and molecular connections between MetS and skin diseases (Figure 2).

#### Adipokines

Adipokines represent a group of hormones and cytokines secreted by adipose tissue, which are widely involved in a variety of biological processes, including energy homeostasis, inflammation, insulin resistance, and cell proliferation (88). Impaired adipose tissue function, such as adipose tissue inflammation, adipocyte hypertrophy, and ectopic fat accumulation, causes an adverse adipose secretion and contributes to metabolic and inflammatory diseases (87). Adipokines can be classified as pro-inflammatory or anti-inflammatory, depending on their effect on inflammation (89). Chemerin, leptin, resistin, and classical cytokines like TNF-α and IL-6, are pro-inflammatory adipokines. Anti-inflammatory adipokines include adiponectin, secreted frizzled-related protein 5 (SFRP5), and omentin-1.

Chemerin has been found to be involved in energy metabolism, adipogenesis, and inflammation. The role of chemerin in body metabolism depends on its role in regulating food intake, glucose homeostasis, and body weight. In adipose tissue, chemerin is involved in the processes of hyperplasia, angiogenesis, and inflammation by targeting adipocytes, endothelial cells, and immune cells, separately. In both mice and humans, chemerin levels are positively correlated with body mass index (BMI), inflammatory cytokines, and percentage of body fat (90, 91). Notably, the expression of chemerin is increased in psoriasis patients and is correlated to PASI (92). A recent study found that chemerin induces the inflammatory response in keratinocytes through reactive oxygen species (ROS)-sirtuin 1 (sirt1)-NFκB signaling (93).

Leptin is mainly produced by adipocytes and is associated with waist circumference, insulin resistance, and MetS (94, 95). Moreover, leptin levels have been found to be higher in psoriasis, Behçet's syndrome, skin tags, and acanthosis nigricans (96–100). The role of leptin in psoriasis is clearly related to its proinflammatory actions. Leptin is reported to induce the secretion of IL-6, TNF-α, IL-1, IL-8, CXCL-1, CXCL-8, and IL-12 (101, 102). These proinflammatory mediators not only lead to insulin resistance and modulate lipid metabolism in MetS, but also contribute to the development of psoriasis. In Behçet's syndrome, apart from the role of leptin in inflammation, leptin also enhances the release of nitric oxide from endothelial cells and is involved in the pathophysiology of vascular lesions (97). In patients with skin tags, serum leptin is significantly upregulated and positive correlations are also found between serum leptin and insulin resistance calculated by homeostasis model assessment (HOMA-IR). Importantly, serum leptin and number of skin tags are significantly higher in patients with MetS compared to patients without MetS (98, 103), indicating that leptin may play a common pathologic role, although the underlying mechanism remains to be elucidated. An immunohistochemical study reported that the angiogenic effect and induction of cellular proliferation of leptin might contribute to the development of skin tags (104).

Resistin is another pro-inflammatory adipokine which is mainly produced by white adipose tissue in mice. In humans, resistin is mainly produced by cell populations such as macrophages, PBMCs, and bone marrow cells (105, 106). The previous data regarding correlation between resistin and MetS or MetS-components was inconsistent (107). However, it has been found that resistin plays an important role in inflammation by increasing the secretion of several inflammatory cytokines including TNF-α, CXCL8, IL-12, IL-1β, and IL-6 (108, 109). Interestingly, resistin has been shown to be associated with psoriasis, AD, AN, and Behçet disease (100, 110–112). Concerning the role of resistin in psoriasis, in addition to its effect on proinflammatory cytokine production, its connection with proprotein convertase subtilisin/kexin 9 (PCSK9) may represent another key mechanism. PCSK9 is a chaperone protein of low-density lipoprotein (LDL), which promotes the degradation of LDL receptors and, therefore, increases the concentration of circulating LDL. PCSK9 inhibitor is already approved to treat hyperlipoproteinemia (113, 114). Our previous study reported that suppressing PCSK9 inhibited the hyper-proliferation of keratinocytes and reduced the psoriasis-like inflammation via the NFκB pathway (115). Interestingly, according to the structure analysis, PCSK9 C-terminal cysteine-rich domain (CRD) reveals structural homology with resistin (116). Moreover, resistin has also been shown to exhibit a positive correlation with PCSK9 levels by enhancing mRNA expression and protein stability (117, 118). As a result, resistin may act as an activator of PCSK9 in the pathogenesis of psoriasis. Regarding its role in AD, resistin is reported to increase the expression of monocyte chemoattractant protein 1 (MCP-1), vascular cell adhesion molecule 1 (VCAM-1), and intercellular adhesion molecule 1 (ICAM-1) in endothelial cells, which contributes to inflammation in AD (116). The expression of resistin was found to be upregulated in AN and Behçet disease, but further studies are still needed to reveal the underlying mechanisms (119, 120).

Adiponectin, as an anti-inflammatory adipokine, has three oligomeric isoforms: a low-molecular-weight trimer, a middle-molecular-weight hexamer, and a high-molecular-weight complex (121). High-molecular-weight adiponectin is reported to be the most biologically active of the three. The circulating levels of adiponectin are decreased in MetS, obesity, and type-2 diabetes mellitus (122). Furthermore, due to its association with severity and incidence of MetS, adiponectin is reported to function as a predictable marker for MetS (123, 124). The major mechanism underlying the relationship between adiponectin and MetS may be the potent insulin-sensitizing effect of adiponectin. Adiponectin can also activate AMPK and PPAR-α pathways via binding to two receptors, including AdipoR1 and AdipoR2 (125). Growing data show that adiponectin is decreased in psoriasis, acne vulgaris, AN, chronic urticaria, and AD (99, 100, 126, 127). It has been suggested that adiponectin suppresses IL-17 synthesis through AdipoR1 and regulates skin inflammation; therefore, it might be a therapeutic target for psoriasis (128). The role of adiponectin in acne vulgaris may be not only related with its anti-inflammatory effects but also associated through its function in inhibiting mTORC1 activity by activating AMP-activated protein kinase (126). In other words, decreased adiponectin levels in acne vulgaris could upregulate activity of the mTORC1 pathway and increase the synthesis of pro-inflammatory cytokines. Although it has been reported that adiponectin is reduced in AN, chronic urticaria, and AD patients, the exact pathogenic role is still not fully understood, which might due to the anti-inflammatory role of adiponectin.

#### Proinflammatory Cytokines Related Molecular Mechanisms

The “inflammatory skin march,” first identified in psoriasis patients with systemic inflammatory condition, might be one of the main mechanisms underlying the connection between MetS and some skin diseases. As for psoriasis, activated myeloid dendritic cells produce IL-12 and IL-23 to promote the development of Th1, Th17, and Th22 cells in psoriasis patients, thus leading to the overproduction of proinflammatory cytokines in psoriatic lesions, including interferon-γ (IFN- γ), IL-1, IL-17, IL-6, IL-12, IL-22, IL-23, and TNF-α (129). These cutaneous inflammatory mediators can migrate into the systemic circulation, which is identified as “inflammatory skin march.” The release of these proinflammatory cytokines can result in chronic systemic inflammation, which induces insulin resistance, obesity, hypertension, and MetS (130, 131). Moreover, atopic dermatitis is a T-cell-mediated disease, with the lesions also characterized by increased expression of cytokines produced by Th2, Th17, and Th22 cells (132). The “inflammatory skin march” is also the reason for the systemic inflammation in AD patients (133). Since the “inflammatory skin march” is reported in psoriasis and AD, it is reasonable to hypothesize that this mechanism could be the common reason for skin inflammation in global inflammatory-related diseases, such as MesS, obesity and, cardiovascular diseases.

Proinflammatory cytokines mediate their effects via binding to their own cytokine receptors and then activating several downstream pathways, including the JAK-STAT pathway, MAPK pathway, and NFκB pathway, of which, the NFκB pathway has been studied thoroughly. The NFκB transcription factor family consists five members, NFκB1 (p105/p50), NFκB2 (p100/p52), RelA (p65), RelB, and c-Rel. The activation of NFκB relies on two major signaling pathways, the canonical and non-canonical pathways, of which, proinflammatory cytokines are associated with the former one, and the non-canonical pathway is associated with differentiation and maturation of immune cells (134). In the canonical NFκB signaling pathway, the dimer of p65 and p50 is in an inactive state by the inhibition of IκB in cytoplasm. Upon stimulation by various cytokine receptors, such as TNF receptor superfamily members, IκB kinase (IKKs) are activated, leading to phosphorylate IκB, which results in their degradation, and enables NFκB translocate to the nucleus to induce the expression of downstream genes, including TNF-α, as well as IL-1, IL-6, and IL-12. The proinflammatory cytokines activate NFκB and then induce the expression of these cytokines, leading to the formation of a feed-forward loop. Recent studies have also shown that NFκB exhibits an anti-inflammatory role, directly inhibiting the expression of inflammatory cytokines by inducing the apoptosis of leukocytes and by upregulating the expression of anti-inflammatory cytokines such as IL-10 (135). Apart from the positive loop, activated NFκB in the nucleus can promote the expression of IκB-α, which binds to and inactivates NFκB in both cytoplasma and nucleus, resulting in a negative feedback loop of the NFκB pathway (136).

The association between NFκB and metabolic syndrome has been studied extensively in obesity and insulin resistance. In obesity, proinflammatory cytokines, free fatty acids, and microbiota-derived LPS can activate IKKβ by directly binding to cytokine receptors and Toll-like receptor 4 in adipocytes, macrophage and muscle (137, 138). The activated IKKβ not only phosphorylates the serine site of IRS, but also activates mTOR and S6K1 by suppressing the TSC1/2 in adipocytes and hepatocytes, both of which contribute to the induction of insulin resistance (139). Besides the inhibition of insulin signaling, activated IKKβ can induce the translocation of NFκB to nuclear by phosphorylating IκB. The activation of NFκB then produces a wide range of chemokines and cytokines in most metabolic tissues, leading to chronic inflammation (138).

In psoriasis, numerous effector cells including keratinocytes, Th17 cells, and dendritic cells activated by TNF-α, plasmin and TLRs could produce cytokines and chemokines via the NFκB pathway, which leads to psoriatic phenotypes (140). Moreover, our recent studies reported that metabolic-associated genes PCSK9 and nuclear receptor interacting protein 1 (NRIP1) both play important roles in psoriasis via the NFκB pathway (115, 141). It has been reported that PCSK9 silencing can suppress atherosclerosis by inhibiting the TLR4/NFκB signaling pathway (142). Also, suppressing PCSK9 can inhibit the NFκB mediated inflammatory response in macrophages (143). In our study, we found that suppressing PCSK9 inhibited the hyper-proliferation of keratinocytes and reduced the RelA/p65-mediated inflammatory reaction induced by imiquimod (115). Likewise, NRIP1, acting as co-activator or co-repressor for several nuclear receptors and transcriptional factors, was also reported to interact with NFκB, thus participating in the inflammatory and metabolic processes. NRIP1 functions as a co-activator in macrophages for NFκB subunit RelA, and cAMP-responsive element binding protein (CREB)-binding protein (CBP), and promotes the TLR-induced production of proinflammatory cytokines such as TNF-α, IL-1β, and IL-6 (144). NFκB (RelA) can inversely resolute the inflammatory response by targeting and degrading NRIP1 via recruiting the SCF (Skp, culin, F-box-containing) E3 ligase complex (145). Our laboratory revealed that suppression of NRIP1 in CD4+ T cells that were isolated from psoriasis patients could downregulate the expression of RelA/p65 and decrease the secretion of IL-17, thus inhibiting the inflammation in psoriasis (141). Importantly, together, PCSK9 and NRIP1 may be two potential therapeutic targets for both psoriasis and metabolic syndrome by regulating the NFκB-associated chronic inflammatory status.

#### Adipose-Derived Mesenchymal Stem Cells (ADMSCs) Therapy in MetS and Skin Diseases

To date, applications of ADMSCs have been studied in a variety of skin diseases, such as alopecia, scars, wound healing, and psoriasis. Although the therapeutic mechanisms of ADMSCs in these diseases remain vague, it has been reported that the reduction of inflammation, the increase of immune cell apoptosis, the promotion of collagen synthesis, and the improvement of angiogenesis might be possibilities (146, 147). It is well known that ADMSCs have the properties of immunomodulatory, which can inhibit inflammation by suppressing the production of proinflammatory cytokines, such as IL-6, IL-17, and TNF-α (148). Moreover, because of the multipotent function, anabolic activity, and immunomodulatory effect, ADMSCs are reported to be a promising therapy for MetS (149). According to the study of MSC transplantation in obese mice, the MSC-based therapies not only reduce obesity-associated metabolic syndromes including non-alcoholic fatty liver disease, non-alcoholic steatohepatitis, glucose intolerance, and inflammation, but also ameliorate high-fat diet-induced obesity and hyperlipidemia (150). A systematic review showed that ADMSCs therapy provides positive effects on body weight, lipid profiles, glucose metabolism homeostasis, non-alcoholic fatty liver disease, and systemic inflammation, which reflects that ADMSCs therapy could be a potential and promising strategy in obesity (151). Furthermore, ADMSCs therapy has been reported in both an imiquimod (IMQ)-induced psoriasis mouse model and in psoriasis patients. In the imiquimod-induced psoriasis mouse model, the intradermal administration of ADMSCs inhibits IL-17A and TNF-a, and suppresses the IMQ-induced inflammation (152). In psoriasis patients (five patients from three case reports), those who received the intravenous injection of ADMSCs, all showed great improvement of psoriatic erythema, scaling, and induration by significant decrease of PASI scores with no severe adverse events (153–155). Considering the chronic inflammatory status in both MetS and psoriasis, along with the results of all these clinical trials, ADMSCs therapy shows great potential to have clinical benefits for patients with combined psoriasis and MetS by reducing inflammation through an immunomodulatory cascade of events (156). Nevertheless, a recent study indicated that mesenchymal stem cells isolated from MetS patients have abnormal apoptosis, autophagy, and mitochondria function, which may limit their therapeutic potential (157). Moreover, mesenchymal stem cells isolated from obese patients have an altered secretome profile such as increased IL-6 and decreased adiponectin (158). Thus, improving the function of the isolated mesenchymal stem cells by genetic treatment may provide a new approach for better clinical outcomes. Several studies have reported that the function of ADMSC can be improved by genetic methods, such as Toll-like receptors depletion, and chemical treatment such as melatonin and transforming growth factor-β (TGF-β) pretreatment (159–161).However, more clinical studies are needed to further evaluate the safety and efficacy of ADMSCs therapy.

## Conclusion

Evidence shows a strong connection between MetS and skin diseases, indicating that some skin diseases may be the cutaneous manifestations of systemic disorders. In this review, we have shown the epidemiological evidence for the connection between MetS and skin diseases, including psoriasis, acne vulgaris, hidradenitis suppurativa, androgenetic alopecia, acanthosis nigricans, and atopic dermatitis. Although the exact relationship between MetS and skin diseases is still unclear, insulin signaling, insulin resistance, and chronic inflammation are believed to contribute. Understanding the mechanisms underlying MetS and skin diseases will help to improve clinical outcomes and guide the development of new therapeutic treatments.

## Author Contributions

YH and NL collected data. YH, YZ, and RY wrote the manuscript. RY, AB, and MC revised the manuscript.

### Conflict of Interest

The authors declare that the research was conducted in the absence of any commercial or financial relationships that could be construed as a potential conflict of interest.
